# Supplementary material for: Introduction to the potential of Ferula ovina in dental implant research due to estrogenic bioactive compounds and adhesive properties
Source: PLoS One. 2022 Jan 18;17(1):e0262045. doi: 10.1371/journal.pone.0262045 (PMC8765653; doi:10.1371/journal.pone.0262045)
Supplement: S1 Fig — Mass Spectrometric (MS) analysis of Ferutinin, Ferula ovina extract and related unknown fraction of TLC: (A) LC-APCI(+) scan of Ferutinin standard (Ferutinin major ion m/z 341.2 = [M+H-H2O]+), (B) MS/MS of Ferutinin Standard (341.2: 203 and 121), (C) MS/MS Ferutinin (Direct infusion of F. ovina extract), (D) LC-MS/MS spectrum of F. ovina extract containing Ferutinin (MRM transitions 341.2: 203.2 represented by blue in spectrum, 121.1 represented by red in spectrum), (E) LC-ESI (+) scan of the unknown fraction of TLC did not present Ferutinin major ion m/z 341.2 = [M+H-H2O]+, (F) LC-ESI (-) scan of the unknown fraction of TLC did not present spectrum as Ferutinin, (G) LC-APCI(+)-MS/MS spectrum of the unknown fraction of TLC, (H) LC-ESI(+)-MS/MS spectrum of the unknown fraction of TLC. (DOCX) [file pone.0262045.s001.docx]

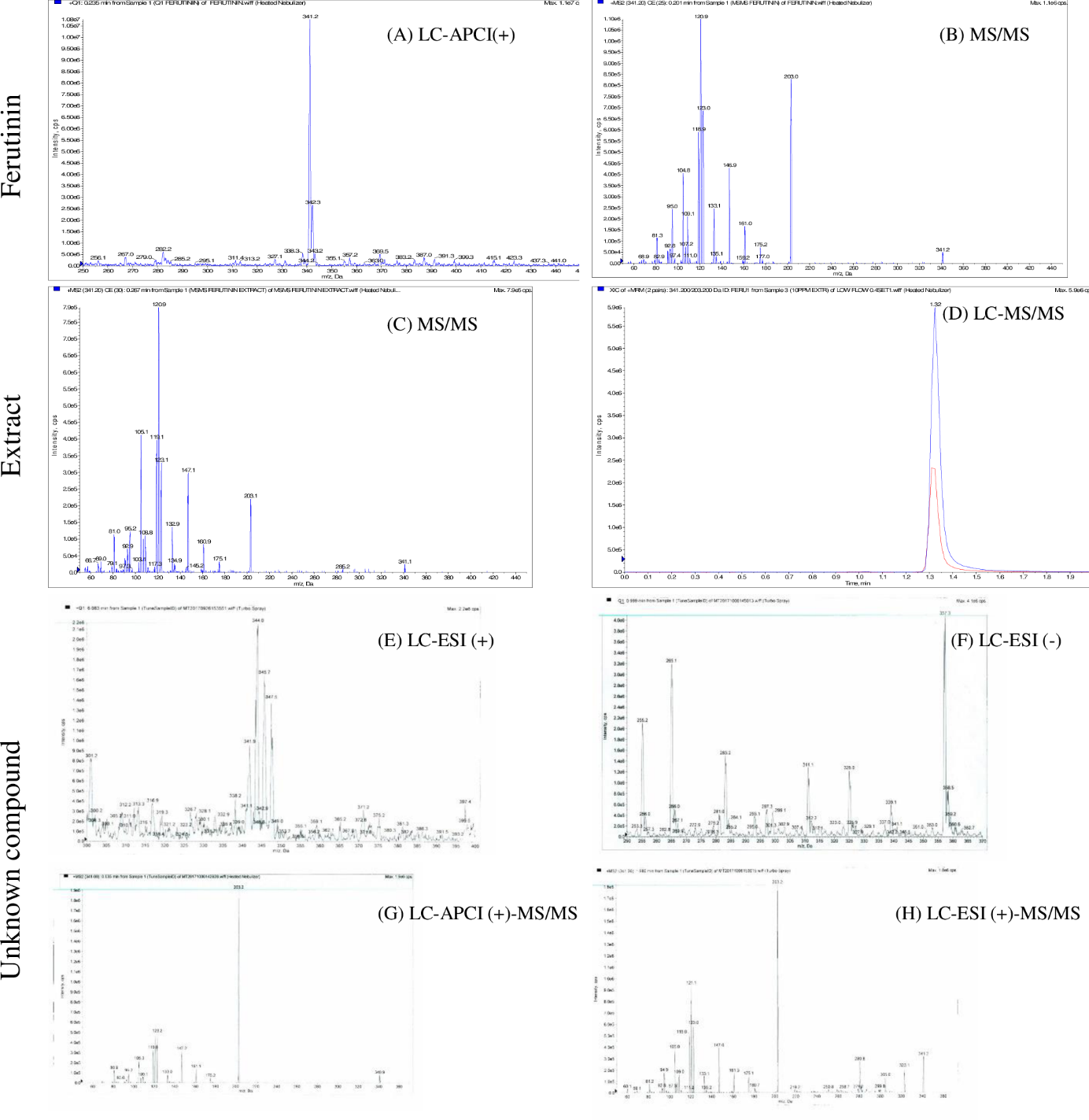


**Supp.1.** Mass Spectrometric (MS) analysis of Ferutinin, *Ferula ovina* extract and related unknown fraction of TLC: (A) LC-APCI(+) scan of Ferutinin standard (Ferutinin major ion *m/z* 341.2 =[M+H-H_2_O]^+^), (B) MS/MS of Ferutinin Standard (341.2: 203 and 121), **(**C) MS/MS Ferutinin (Direct infusion of *F. ovina* extract), (D) LC-MS/MS spectrum of *F. ovina* extract containing Ferutinin (MRM transitions 341.2: 203.2 represented by blue in spectrum, 121.1 represented by red in spectrum), **(**E) LC-ESI (+) scan of the unknown fraction of TLC did not present Ferutinin major ion *m/z* 341.2 = [M+H-H_2_O]+, (F) LC-ESI (-) scan of the unknown fraction of TLC did not present spectrum as Ferutinin, (G) LC-APCI(+)-MS/MS spectrum of the unknown fraction of TLC, (H) LC-ESI(+)-MS/MS spectrum of the unknown fraction of TLC .
