# Supplementary figures and images for: Introduction to the potential of Ferula ovina in dental implant research due to estrogenic bioactive compounds and adhesive properties
Source: PLoS One. 2022 Jan 18;17(1):e0262045. doi: 10.1371/journal.pone.0262045 (PMC8765653; doi:10.1371/journal.pone.0262045)

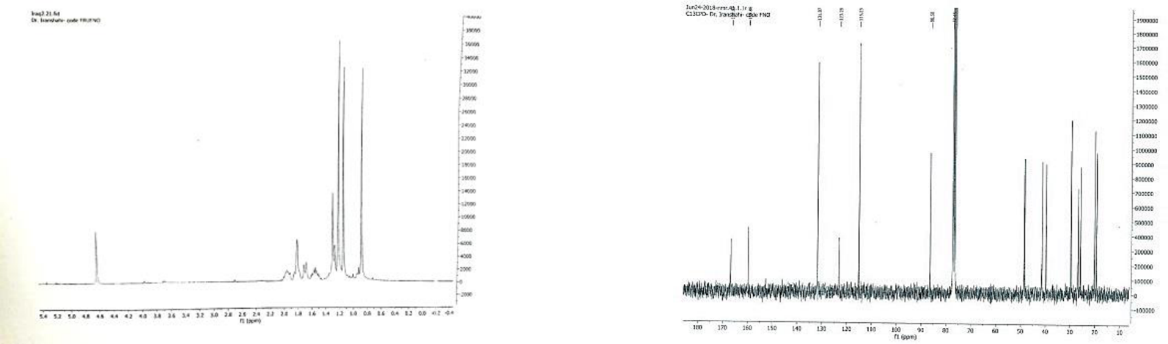


**Supp.2.** NMR of an unknown compound isolated from root extract of *Ferula ovina*.

Supplement: S2 Fig — (DOCX) [file pone.0262045.s002.docx]

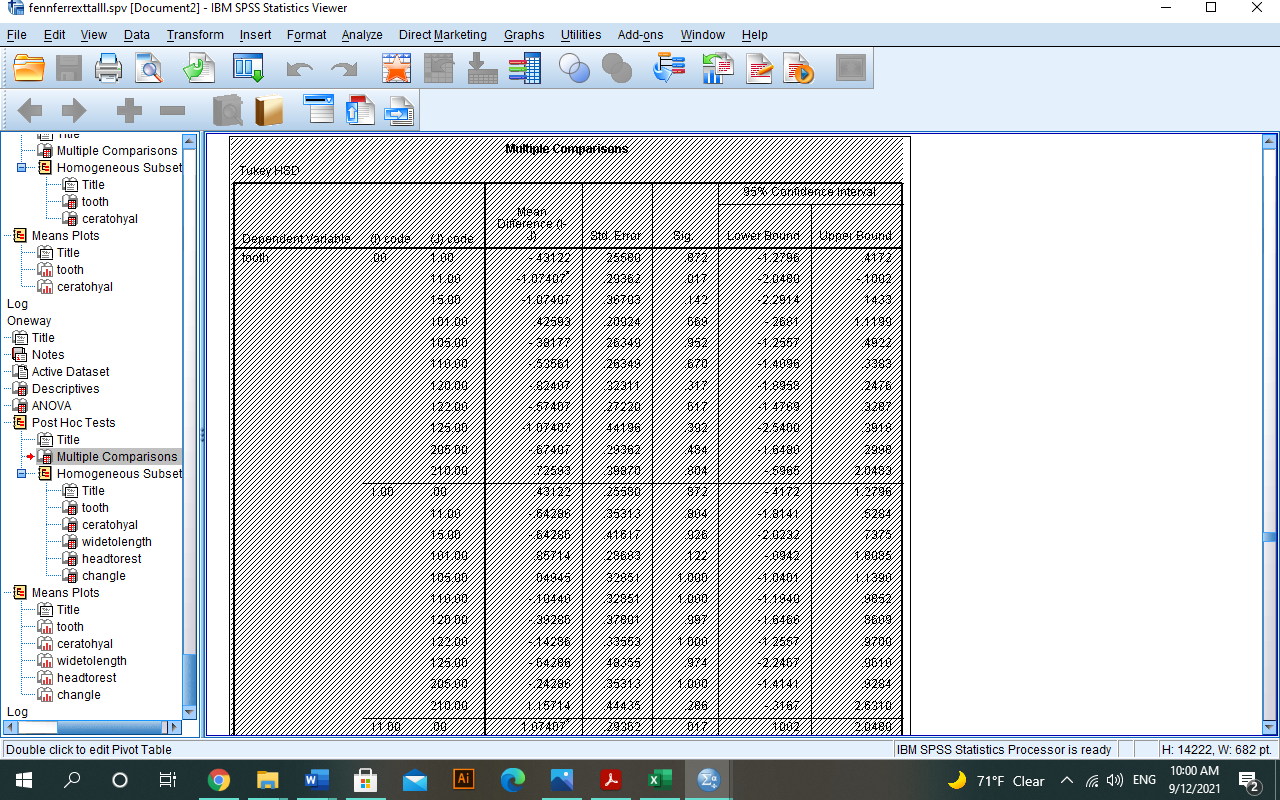

Supplement: S3 Fig — (PNG) [file pone.0262045.s003.png]

**Graphical abstract**


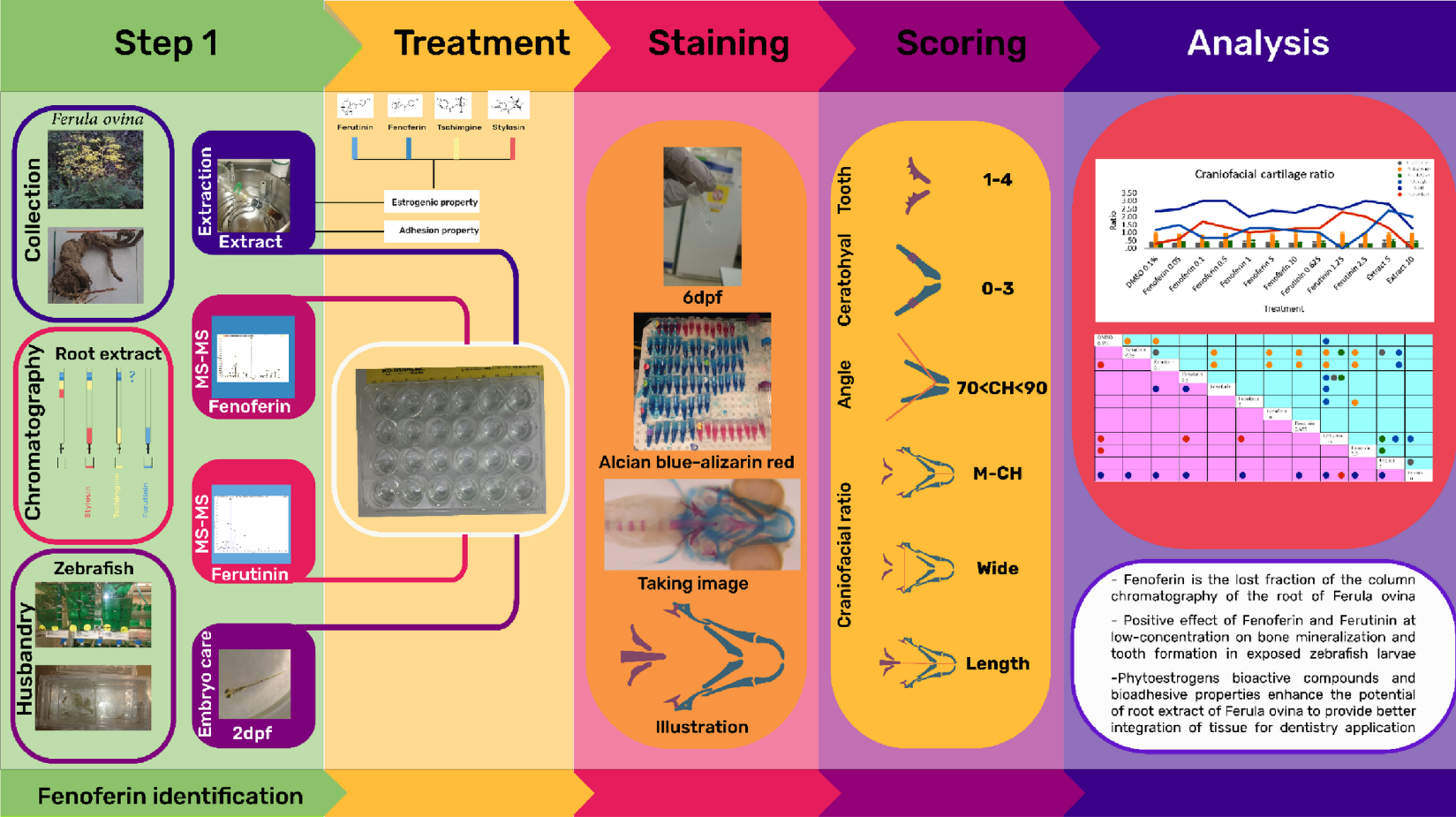

Supplement: S1 Graphical abstract — (DOCX) [file pone.0262045.s006.docx]
