# Supplementary material for: Introduction to the potential of Ferula ovina in dental implant research due to estrogenic bioactive compounds and adhesive properties
Source: PLoS One. 2022 Jan 18;17(1):e0262045. doi: 10.1371/journal.pone.0262045 (PMC8765653; doi:10.1371/journal.pone.0262045)
Supplement: S1 File — (PDF) [file pone.0262045.s005.pdf]

Certificate of Training

This certifies that

Hoda Zare Mirakabad

has successfully completed the following course presented by
Research Services and Ethics:

UACC Animal Ethics Course: Lab Animals

Instructor: Dr Corinna Kashuba

UNIVERSITY OF SASKATCHEWAN

16 April 2017
