# Supplementary material for: Introduction to the potential of Ferula ovina in dental implant research due to estrogenic bioactive compounds and adhesive properties
Source: PLoS One. 2022 Jan 18;17(1):e0262045. doi: 10.1371/journal.pone.0262045 (PMC8765653; doi:10.1371/journal.pone.0262045)
Supplement: Attachment — Submitted filename: Response to Reviewer 1.docx [file pone.0262045.s007.docx]

Response to Reviewer 1 Comments

**Point 1:** The paper is poor, preliminary and badly written and organized.

**Response 1:** The current paper was rewritten and excluded the sections made it poor, preliminary and badly written and organized

**Point 2:** English language should be revised by a native speaker.

**Response 2:** English language was revised by a native speaker.

**Point 3:** Introduction section should be completely rewritten: it is too long and confusing and presented as a list of results of other studies.

**Response 3:** Introduction section was completely rewritten. According to the aim of the current study, each paragraph of the introduction section was necessary to make a right view.

**Point 4:** It is not clear the aim of the study.

**Response 4:** The aim of the study is quantification of the effect of different phytoestrogens derived from *F. ovina* on craniofacial calcification that was inferred clearly in the last paragraph of introduction.

**Point 5:** The results are badly organized and difficult to understand! No in vitro analysis of the effect of the substance on osteogenic differentiation was done.

**Response 5:** I tried to organize the results as well as possible. The current paper is based on the research that I performed at usask and in vitro analysis of the effect of the substance on osteogenic differentiation had not been defined.

**Point 6:** Authors aimed to demonstrate the effect of Fenoferin to treat osteoporosis, but it is not clear what effect this substance has on osteogenesis.

**Response 6:** Firstly, I should refer to the point 4 of reviewer,” It is not clear the aim of the study.” Secondly, the analysis of changing in bone mineralization using alcian blue- alizarin red staining indicated effect of substance on craniofacial calcification. Moreover, the manuscript has been rewritten, completely.

**Point 7:** Authors should perform experiments on gene and protein expression of osteogenic markers.

**Response 7:** As I inferred in response 5, regard to the project that was defined at usask, performing experiments on gene and protein expression of osteogenic markers were not necessary and we followed the changes in phenotype.

**Point 8:** Discussion section should be rewritten as a single paragraph.

**Response 8:**  It was not possible to rewrite discussion section as a single paragraph.

**Point 9:** Conclusions are overinterpreted.

**Response 9:** The current paper was rewritten and excluded the conclusions sections.
